# Detoxification Gene Families at the Genome-Wide Level of *Rhus* Gall Aphid *Schlechtendalia chinensis*

**DOI:** 10.3390/genes13091627

**Published:** 2022-09-10

**Authors:** Hongli He, M. James C. Crabbe, Zhumei Ren

**Affiliations:** 1 School of Life Science, Shanxi University, Taiyuan 030006, China; 2 Wolfson College, Oxford University, Oxford OX2 6UD, UK; 3 Institute of Biomedical and Environmental Science & Technology, University of Bedfordshire, Luton LU1 3JU, UK

**Keywords:** Aphididae, *Schlechtendalia chinensis*, detoxification gene family, genome-wide, identification

## Abstract

The *Rhus* gall aphid *Schlechtendalia chinensis* uses the species *Rhus chinensis* as its primary host plant, on which galls are produced. The galls have medicinal properties and can be used in various situations due to their high tannin content. Detoxification enzymes play significant roles in the insect lifecycle. In this study, we focused on five detoxification gene families, i.e., glutathione-S-transferase (GST), ABC transporter (ABC), Carboxylesterase (CCE), cyto-chrome P450 (CYP), and UDP-glycosyltransferase (UDP), and manually annotated 144 detoxification genes of *S. chinensis* using genome-wide techniques. The detoxification genes appeared mostly on chromosome 1, where a total of two pair genes were identified to show tandem duplications. There were 38 gene pairs between genomes of *S. chinensis* and *Acyrthosiphon pisum* in the detoxification gene families by collinear comparison. Ka/Ks ratios showed that detoxification genes of *S. chinensis* were mainly affected by purification selection during evolution. The gene expression numbers of P450s and ABCs by transcriptome sequencing data were greater, while gene expression of CCEs was the highest, suggesting they might be important in the detoxification process. Our study has firstly identified the genes of the different detoxification gene families in the *S. chinensis* genome, and then analyzed their general features and expression, demonstrating the importance of the detoxification genes in the aphid and providing new information for further research.

## 1. Introduction

The *Rhus* gall (or sumac-gall) aphids switch host plants between *Rhus* species and mosses to finish their life cycles, and form galls on their primary host plants *Rhus* (Anacardiaceae) species [1,2,3]. The galls are often known as the Chinese galls and they are rich in tannins and economically important in Asia because galls have medicinal properties and represent sources of industrial tannin [4,5]. This aphid group belonged to the subtribe Melaphidina of tribe Fordini (Aphididae: Eriosomatinae) [6,7,8], including six genera and 12 species [3], among which *S. chinensis* is the most common and wide-spread species, with *R. chinensis* as its unique primary host plant and Mniaceae species as its secondary hosts, as well as a life cycle including both sexual and asexual reproduction stages [9,10].

Phytophagous insects and host plants have coevolved, and plants have evolved physical or chemical defense mechanisms to resist insect feeding, while insects have evolved perfect anti-defense mechanisms [11]. Insects adapted to host plants partly rely on their detoxification genes, whose mechanism has been divided into three stages, i.e., an initial oxidation/reduction-hydrolysis involving mainly cytochrome P450 monooxygenase (P450) and carboxylesterases (CCE), enzymatic conjugation UDP-glucuronyl transferases (UGTs) or glutathione S-transferases (GST), and conjugated-metabolite transport-excretion out of the cells (ABC transporters (ABC)) [12,13,14].

The P450 gene family (P450s) has the ability to diminish the biological activity of a wide range of endogenous toxic compounds and exogenous substances [15]. Thomas et al. (2021) annotated 66 cytochrome P450s in Phylloxera *Daktulosphaira vitifoliae* and classified them into four clades [16]. Carboxylesterase (CCEs) belongs to one gene family of the α/β hydrolase protein superfamily [17], and fifty-seven putative CCEs was identified in *Anopheles sinensis* and divided into three classes, 12 subfamilies and 14 clades [18]. Glutathione S-transferases (GSTs) and uridine diphosphate-glycosyltransferases (UGTs) are conjugation enzymes, that covalently attach small endogenous hydrophilic molecules in order to increase compound solubility and facilitate their excretion, which have effects on the toxic by-products of phase I metabolism [19]. Thirty-six putative cytosolic GSTs and five microsomal GSTs were identified in *Tribolium castaneum* to reveal the largest insect-specific GSTs: Epsilon and Delta [20]. ATP-binding cassette transporters (ABC) transport substrates including amino acids, lipids, peptides, sugars and drugs across cell membranes by using ATP hydrolysis energy [21]. A total of 47 ABC genes in *Bactrocera dorsalis* were identified and classified into eight subfamilies (A–H), and it was suggested that these genes may play important roles in xenobiotic metabolism and biosynthesis in *B. dorsalis* [22].

However, there is no report on the detoxification genes in the *Rhus* gall aphid *S. chinensis* genome. Here, we used the third-generation sequencing technology to obtain the whole genome of *S. chinensis* at the chromosome level and performed the comprehensive analysis of five detoxification gene families in the *S. chinensis* genome. In detail, we conducted systematic identification and molecular characterization, which included gene family member identification, collinear analysis, chromosomal location, Ka/Ks evolutionary selection pressure analysis, gene expression analysis, protein physicochemical properties, and structure prediction. We highlight the characters of the key detoxification genes in *S. chinensis-R. chinensis* adaptive interactions for future functional studies.

## 2. Materials and Methods

### 2.1. Sample Information

Mature and fresh *Rhus* galls were collected from the garden in Shanxi University, Shanxi, China. The gall was cut open and live aphids were used for third-Generation Sequencing. In addition, we selected the aphid samples from three mature galls, numbered A4601, A4603, and A4621 for transcription sequencing to characterize the gene expression pattern of detoxification genes in *S. chinensis*. The specimens were stored at the School of Life Science, Shanxi University, China.

### 2.2. Identification of Detoxification Genes from S. chinensis

We sequenced the whole genome of *S. chinensis* by third—generation sequencing on the Pacbio platform. The protein-coding genes in the genome were annotated by integrating three approaches, namely de novo prediction, homology search, and transcript-based assembly. The protein sequences of the detoxification genes were obtained by searching in the annotation table of *S. chinensis* using key words of detoxification genes, and then confirmed in the NCBI Conserved Domain Database (NCBI-CDD) (e-value = 1 × 10^−3^), excluding those lacking conserved domains from the analysis [23].

### 2.3. Phylogenetic Tree, Motif Pattern, Domain, Gene Structure of Detoxification Genes

The protein coding sequences of detoxification genes of the three aphid species, *A. pisum*, *Cinara cedri* and *Myzus persicae*, were downloaded from the Insect BASE website (http://v2.insect-genome.com/, accessed on 12 May 2022). We performed the multiple-sequence alignment of detoxification genes using ClustalW software (created by Kumar et al.; Philadelphia, PA, USA) [24]. The alignment results were exported to fasta format, and then opened using tbtools to trim using Trimmer [25]. The protein sequences with large differences were filtered out. We constructed a phylogenetic tree using the Neighbor-Joining (NJ) method with the parameters of Poisson model, complete deletion and 1000 bootstrap replicates, and visualized and improved the tree using the program Evolview (http://www.evolgenius.info/evolview/, accessed on 18 March 2022) [26]. The relative frequency of the corresponding amino acid at each position was calculated on the Web Logo online website (http://weblogo.threeplusone.com/, accessed on 18 March 2022). We conducted motif analysis of the detoxification genes on the MEME online server (http://meme-suite.org/tools/meme, accessed on 20 March 2022) with parameters “minimum width = 6, maximum width = 50, number of motif to find = 10” [27], and the analysis of conserve domain by the NCBI Conserved Domain Database (NCBI-CDD) (e-value = 1 × 10^−3^) [23]. The exon and intron structures were displayed in all protein sequences using the Gene Structure Display Server (GSDS) (http://gsds.cbi.pku.edu.cn/, accessed on 22 March 2022) [28]. Finally, the corresponding results were visualized by the program TBtools [29].

### 2.4. Chromosomal Locations, Collinearity and Selection Pressure

The detoxification genes’ positions on chromosomes were displayed using TBtools (version 1.09876, created by Chen et al.; Wuhan, China) [29]. We conducted and visualized collinearity analysis by MCScanX (created by Wang et al.; Wuhan, China) [30] and Circos (version 2.50, created by Krzywinski et al., Vancouver, Canada) [31]. In addition, we used Ka Ks_Calculator 2.0 (version 2.0, created by Zhang et al.; Beijing, China) to calculate the ratio between the non-synonymous replacement rate (Ka) and the synonymous replacement rate (Ks) of two protein-coding genes, which is an indicator of molecular evolution to determine whether there is selective pressure on a protein-coding gene [32].

### 2.5. Expression Profile of Detoxification Genes

We extracted total mixed RNA from the three *S. chinensis* samples by the Trizol method [33], and constructed a sequence library using the Illumina TruseqTM RNA sample prep Kit [34]. On the Illumina HiSeq2500 platform, we carried out next generation sequencing for the sequence library. We measured the original raw reads and removed the low-quality, repetitive data with adapters to obtain clean reads. Finally, we assembled the transcriptome data from scratch to obtain the unigenes sequence by the Trinity assembly software [35]. Meanwhile, we compared unigenes sequence with six major databases, i.e., NR, Swiss-Prot, Pfam, COG, GO, and KEGG, to complete functional annotation and classification analysis [36,37,38,39,40,41,42,43]. We screened detoxification-related genes with our gene annotation lists. We compared the CDS sequences of the genomic detoxification genes with the transcriptome detoxification gene by MAFFT Alignment to determine the gene expression [44].

### 2.6. Prediction of Characteristics and Physicochemical Properties of Detoxification Gene

We used the online bioinformatics software Expasy Protoparam (https://web.expasy.org/protparam/, accessed on 23 March 2022) to predict the protein length, molecular weight, and isoelectric point of the keratins [45], and Signal P 5.0 Server (http://www.cbs.dtu.dk/services/SignalP/index.php, accessed on 24 March 2022) and TMHMM Server v.2.0 (https://services.healthtech.dtu.dk/service.php?TMHMM-2.0, accessed on 24 March 2022) to predict signal peptides, respectively [46,47].

### 2.7. Protein Structure Prediction from the Detoxification Genes

We analyzed the protein secondary structures of the detoxification gene products of *S. chinensis* by the program SOPMA with the website (https://npsa-prabi.ibcp.fr/cgi-bin/npsa_automat.pl?page=/NPSA/npsa_sopma.html, accessed on 24 March 2022) [48]. We predicted the tertiary structure of *S. chinensis* by Phyre2 (http://www.sbg.bio.ic.ac.uk/phyre2/html/page.cgi?id=index, accessed on 24 March 2022) [49].

## 3. Results

### 3.1. Identification of Detoxification Genes of S. chinensis

We identified nine genes in GSTs, 55 genes in ABCs, 18 genes in CCEs, 48 genes in CYPs and 14 genes in UDPs in the *S. chinensis* genome (Table 1). These genes were divided into three subfamilies for GSTs, i.e., delta, theta, and sigma, six subfamilies for ABCs, i.e., A, B, C, D, E, F, and G, four subfamilies for P450s, i.e., CYP2, CYP6, CYP4, and mitochondrion, two subfamilies UGT4 and UGT5 for UDPs. Detoxification gene numbers displayed heterogeneity in the annotated Aphidinae genomes. For example, *A. pisum* displayed more genes of GSTs than other aphid species due to having more genes in delta subfamily. *S. chinensis* had the greatest numbers in ABCs due to more genes in the C subfamily, and fewer in CCEs and CYPs due to fewer genes in Esterase and CYP6 subfamilies, respectively. These gene families, i.e., GSTs, ABCs, and CCEs, clearly exhibited expansion and contraction.

### 3.2. Characteristic of the Five Detoxification Genes of S. chinensis

We performed a characteristic analysis of the detoxification genes, including motif, domain, and the number of exons, and constructed the phylogenetic tree of the protein sequences of five detoxification gene families from the four aphid species *S. chinensis*, *A. pisum, C. cedri,* and *M. persicae*.

#### 3.2.1. P450s

**Figure 1:**
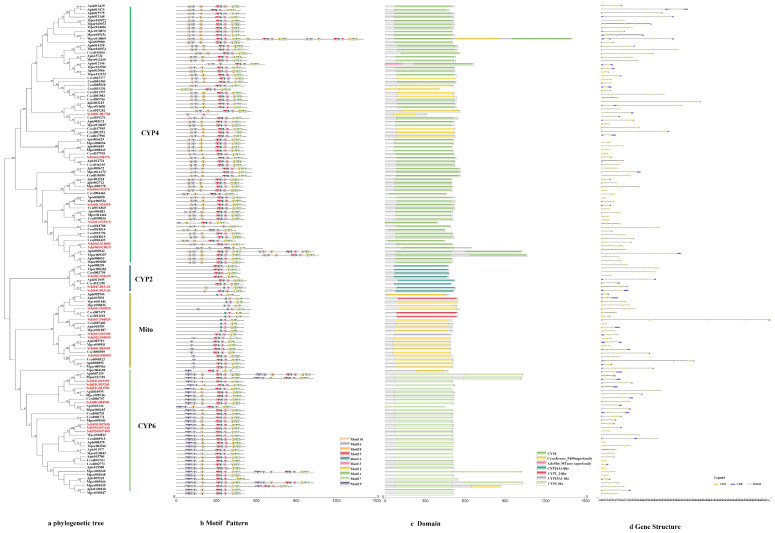
(**a**) Phylogenetic relationships, (**b**) conserved motifs, (**c**) domains and (**d**) gene structure analysis of detoxification genes of the P450 gene family in *S. chinensis*, *A. pisum*, *C. cedri*, and *M. persicae*.

**Figure 2:**
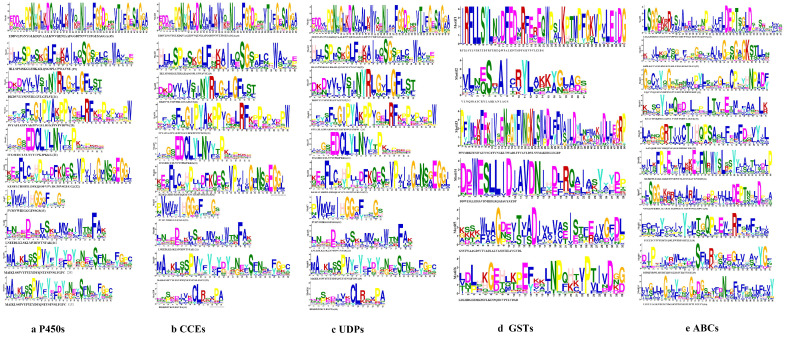
The 10 conserved motifs of detoxification genes families in the *S. chinensis*. (**a**) P450 gene family. (**b**) CCE gene family. (**c**) UDP gene family. (**d**) GST gene family. (**e**) ABC gene family.

The phylogenetic tree of the P450s divided the sequences into four subfamilies, i.e., CYP4, CYP2, mitochondrial clan and CYP6 (Figure 1a). The genes in the same class had similar motif patterns and domain. For example, most of the motif order of *S. chinensis* in the CYP4 class were 10-6-8-5-4-3-2-1-7, except for Schi08G001700 with 6-3. All the CYP2 class motif was 5-4-3-2-1-7. The mitochondrial clan motif order was different with 4-3-2-1, 5-4-3-2-1 or 6-5-4-3-2-1-7. The CYP6 class motif order was 9-6-8-5-4-3-2-1-7 (Figure 1b). The length of ten conserved motifs of P450s varied from 15 to 50 amino acids (Figure 2a). The conserved domain included CYP4, cytochrome_P450, AdoMet_MTases, CYP24A1, CYP1_2, CYP15A1 and CYP6 (Figure 1c). The numbers of exons ranged from three to 22 from predictions of the gene structure (Figure 1d). Gene length varied from 0 to 61kb in the P450s, among which the majority (65%) were 0–10 kb, and a small proportion (35%) of the genes were greater than 10 kb in size.

#### 3.2.2. CCEs

**Figure 3:**
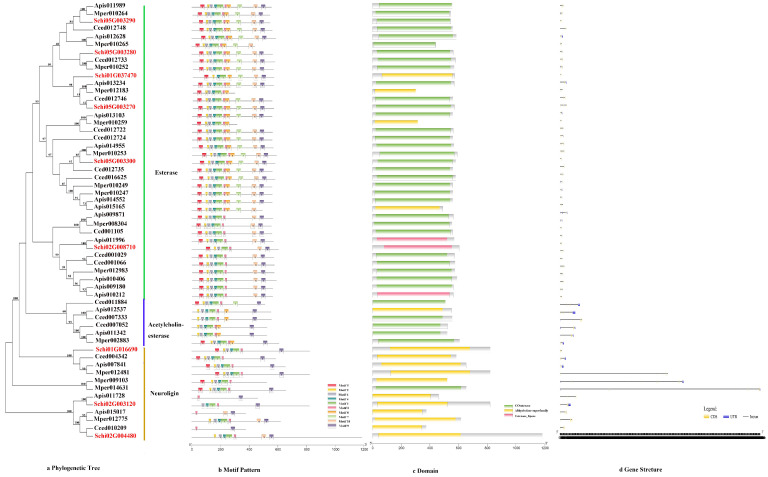
(**a**) Phylogenetic relationships, (**b**) conserved motifs, (**c**) domains and (**d**) gene structures of detoxification genes of the CCE gene family in *S. chinensis*, *A. pisum*, *C. cedri*, and *M. persicae*.

The phylogenetic tree of the CCEs divided the detoxification genes into three subfamilies, i.e., Esterase, Acetylcholinesterase and Neuroligin (Figure 3a). The motif order of the three classes was different, but most of them had the common order 2-6-4-1-3-8-7-10-9 (Figure 3b). Ten conserved motifs of CCEs varied from 15 to 45 amino acids in length (Figure 2b). The conserved domain of CCEs included coesterase, Abhydrolase and esterase _lipase (Figure 3c). The analysis on the coding sequence (CDS) and untranslated regions (UTRs) of the CCEs showed that exon numbers ranged from two to 18, and a total of 41 members exhibited 5′and 3′ UTRs, eight members presented no UTR and the remaining seven members had either a 5′or 3′ UTR (Figure 3d). Differences in the motif pattern and gene structure of the different classes might be the reason for the differences in their physiological functions.

#### 3.2.3. UDPs

**Figure 4:**
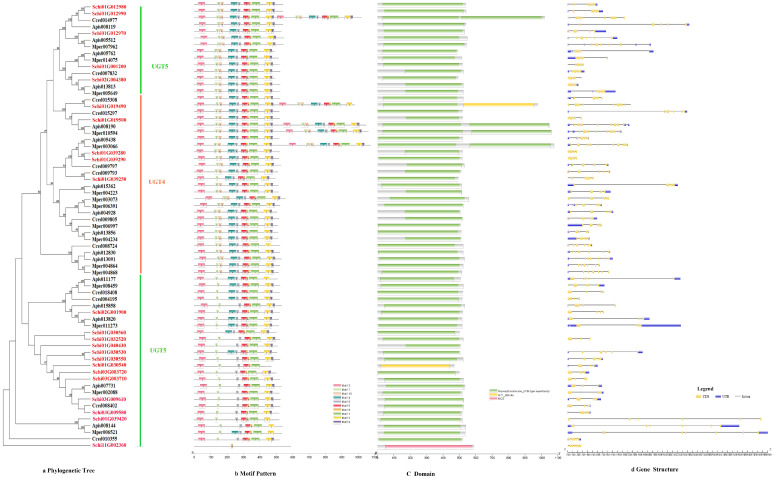
(**a**) Phylogenetic relationships, (**b**) conserved motifs, (**c**) domains and (**d**) gene structures of detoxification genes of the UDP gene family in *S. chinensis*, *A. pisum*, *C. cedri*, and *M. persicae*.

The phylogenetic tree divided the protein sequence of UDPs into two subfamilies, i.e., UGT4 and UGT5 (Figure 4a). All genes of UDPs showed the same motif order 3-7-10-4-6-5-8-1-2-9, only few genes lacked a 1-2 motif, i.e., Schi02G001900, Schi01G030560 (Figure 4b). Ten conserved motifs of UDPs varied from 15 to 50 amino acids in length (Figure 2c). The conserved domain of UDPs included Glycosyltransferase _GTB, GT1_Gtf and MCS (Figure 4c). Analysis of the coding sequence (CDS) and untranslated regions (UTRs) of the UDPs showed that the numbers of exons ranged from three to 11. Only two genes had 11 exons, 27 UDP genes (17.3%) had five exons, and 25 UDP genes (81.7%) had four exons. A total of 43 members exhibited 5′and 3′ UTRs, 13 members presented no UTR, and the remaining five members had either 5′or 3′UTR. The result shows that the same class has the same number of exons in the UDP gene family (Figure 4d).

#### 3.2.4. GSTs

**Figure 5:**
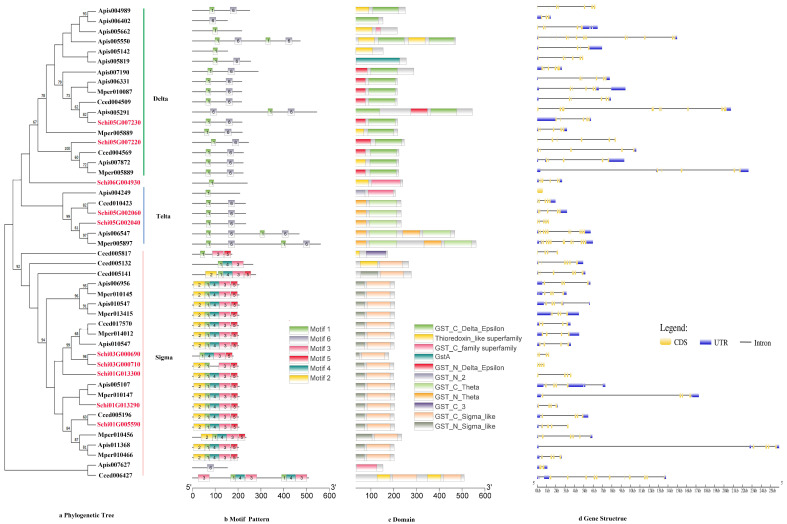
(**a**) Phylogenetic relationships, (**b**) conserved motifs, (**c**) domains and (**d**) gene structures of detoxification genes of the GST gene family in *S. chinensis*, *A. pisum*, *C. cedri*, and *M. persicae*.

The phylogenetic tree divided the protein sequence of GSTs into three class, i.e., Delta, Theta and Sigma (Figure 5a). The delta subfamily included four genes, i.e., Schi05G007220, Schi05G002040, Schi05G002060, and Schi05G007230, which shared the same motif order (1-6) as the nine genes in the Sigma subfamily. Schi01G005590, Schi01G013290, Schi01G013300, Schi03G000690 and Schi03G000710 belonged to theta subfamily and shared the same motif order (3-1-4-2-5) (Figure 5b). Details of six putative motifs are outlined in Figure 2d. These conserved motifs ranged from 15 to 50 amino acids in length. The conserved domain of GSTs included GST _ Delta _ Epsilon, Gst A, GST _Theta, GST _Sigma and Thioredoxin (Figure 5c). In the delta and theta subfamily, the numbers of most exons were four, a few exons were just one. In the sigma subfamily, the numbers of most exons were 11, and other members had five exons (Figure 5d). The genes in the same groups had similar motif patterns and numbers of exons, indicating that they were highly conserved and the inferred functions were similar.

#### 3.2.5. ABCs

**Figure 6:**
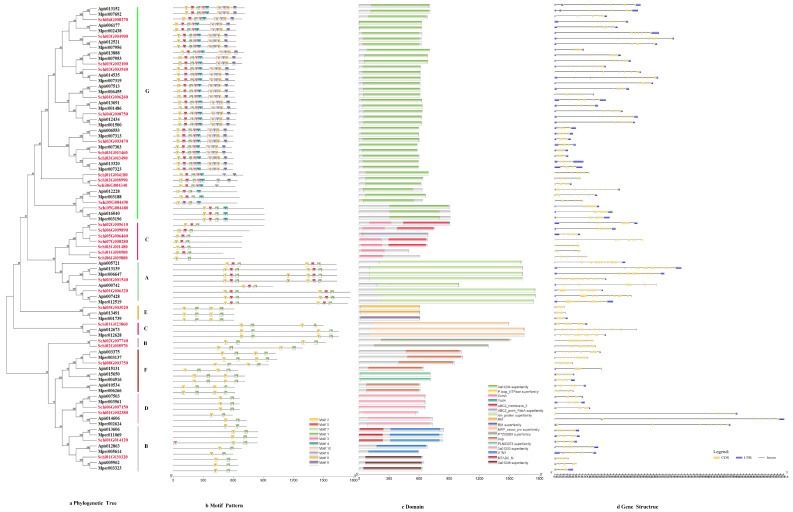
(**a**) Phylogenetic relationships, (**b**) conserved motifs, (**c**) domains and (**d**) gene structures of detoxification genes of the ABC gene family in *S. chinensis*, *A. pisum*, *C. cedri*, and *M. persicae*.

The phylogenetic tree of the protein sequences of ABCs of *S. chinensis* were distributed to G, C, A, E, C, B, F and D subfamilies (Figure 6a). The motif order for G class was 2-5-7-1-3-4-10-6-8-9. The C class motif order was 2-5-7-1. A class motif order for most members was 2-5-7-1, with a few members (Schi03G001540) having special motif 8. The E, C, B, F and D class motif order was 2-7-1 (Figure 6b). The lengths of ABC conserved motifs ranged from 28 to 41 amino acids (Figure 2e). The conserved domain of ABCs included 3a1204, CcmA, YadH, Rli1, Uup, ATM1, MTABC and ABC2_membrane_3 (Figure 6c). The numbers of exon ranged from six to 28 by the predictions of the gene structure. The exon number of most members was 14-28. Analysis of the coding sequence (CDS) and untranslated regions (UTRs) of the ABC gene family found that just six members did not have a UTR (Figure 6d).

### 3.3. Chromosomal Location and Collinearity of Detoxification Genes of S. chinensis

**Figure 7:**
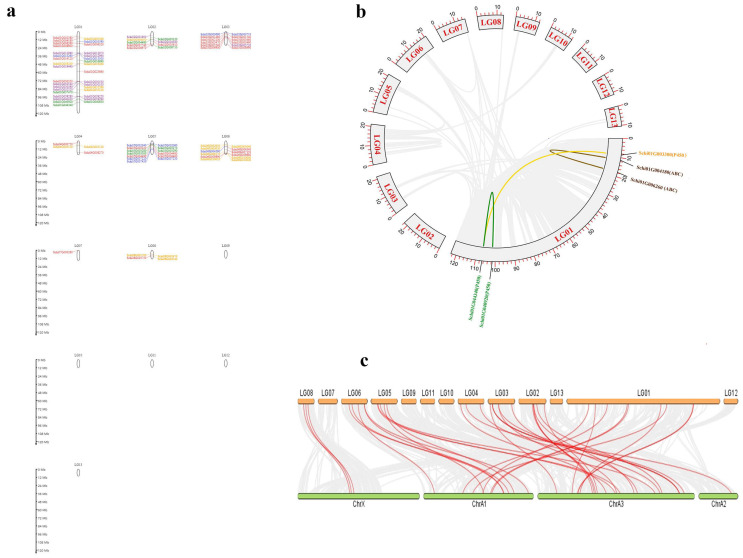
Location and collinearity analysis of all detoxification genes in *S. chinensis*. (**a**) Scaffold location and gene tandem. Green represent CCEs; Blue represent GSTs; orange represent P450s; purple represent UDPs; Red represent ABCs (**b**) Chromosomal location and collinearity. Grey boxes represent chromosomes. lighted lines connected detoxification gene duplication. (**c**) Synteny on gene families of *S. chinensis* and *A. pisum*.

The location and collinearity analysis of all detoxification genes showed that 144 genes were unevenly distributed on chromosomes 1–9 (Figure 7a). Chromosome 1 had the most members of detoxification genes with 33 genes, among which there were eight genes in ABCs, four genes in CCEs, three genes in GSTs, six genes in P450s and 12 genes in UDPs. A total of two pair genes showed tandem duplications on Chromosome 1. The two genes Schi01G040920 and Schi01G044340 of the P450 family, and Schi01G004180 and Schi01G00620 of the ABCs showed tandem duplications, respectively. Only two pairs of genes in the detoxification genes had collinearity (Figure 7b). Chromosome 7 had the fewest detoxification genes with only one member. The distribution of detoxification genes on chromosomes with no bias to the 5′ or 3′ ends may be related to their function.

The collinear comparison map of the detoxification gene family was established by MC Scan X between *S. chinensis* and *A. pisum* (Figure 7c). There were 38 pairs of collinearity (homologous gene pairs) in *A. pisum*, including nine in P450s, 16 in ABCs, three in CCEs, three in GSTs, and seven in UDPs. There were more homologous gene pairs of ABCs and P450s in the *S. chinensis* and *A. pisum*, which may be related to the large number of these two gene families. It is inferred that these two gene families are relatively conservative in the evolutionary process and have relatively stable functions.

The Ka/Ks analysis of 38 pairs of homologous genes existing in *S. chinensis* and *A. pisum* was carried out, and the results are shown in Table 2. The ratios of Ka/Ks between gene pairs were all <1, which indicated that the detoxification genes in *S. chinensis* were mainly affected by purification selection during evolution.

### 3.4. Expression Profiles of Detoxification Genes in S. chinensis

We examined 61 detoxification genes in the transcriptome data of *S. chinensis*, among which there were 13 genes in ABCs, nine genes in CCEs, seven genes in GSTs, 19 genes in P450s and 11 genes in UDPs, respectively. The gene number expressed in the P450s was the highest and the gene number expressed in GSTs was the least (Table 3). In terms of gene expression, the overall expression of CCEs was the highest, while that of P450s was the lowest. Individual gene expression was particularly high in some gene families. For example, the expression level of Schi01G005590 (GSTs) was 251.68, Schi01G003300 (P450s) was 166.92., and Schi05G003270(CCEs) was 127.19, from which we infer that these genes play an important role in the detoxification process of *S. chinensis.*

### 3.5. Physicochemical Properties of Detoxification Gene Products in S. chinensis

The molecular weight of the detoxification genes products ranged from 20,632.58 Da to 199,951.12 Da (Table 4), the number of amino acids from 178 to 1764, and the aliphatic index from 74.78 to 112.86. The isoelectric points (pI) ranged from 4.93 to 9.58. The instability index of which 51% of the detoxification gene products were more than 40, indicated that those genes were unstable and easily degraded. The aliphatic amino acid index of detoxification gene proteins was 74.78–112.86, and the average of the hydropathicity indices ranged from −0.81 to 0.306. The proteins with hydropathicity value less than 0 were hydrophilic proteins.

### 3.6. Prediction of Protein Multi-Level Structures of Detoxification Gene Products in S. chinensis

**Figure 8:**
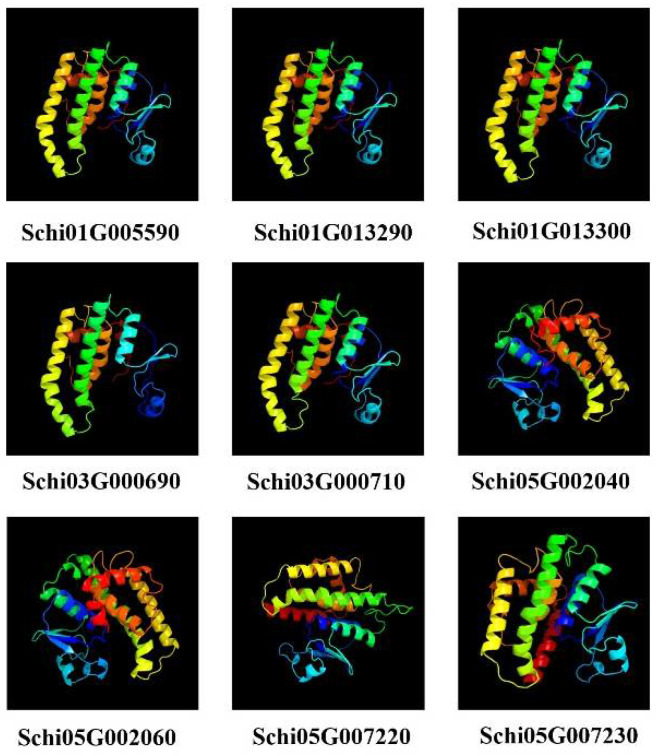
Prediction of tertiary structures of GSTs proteins in *S. chinensis*.

Since the members of detoxification gene family products have similar protein structures, we selected the protein members of GSTs to predict secondary and tertiary structures. The predicted secondary structures of the nine GST proteins are shown in Table 5. The GST protein was composed of four parts: α-helix, extended chain, β-turn and random coil, among which the α helix ratio of the GST protein was the highest, followed by random coil, and the ratio of β-turn was the smallest. Our Signal P prediction showed that there were no signal peptides in all the GST proteins of *S. chinensis.* The tertiary structures of GST proteins are shown in Figure 8. Schi01G005590, Schi01G013290, Schi01G013300, Schi03G000690 and Schi03G000710, Schi05G002040 and Schi05G002060, Schi05G007220, and Schi05G007230 have similar protein structures, respectively. These genes belonged to sigma, theta, and delta subfamilies, respectively. The result indicated that gene members belonging to the same subfamily have similar protein structures, indicating they have similar biological functions.

## 4. Discussion

### 4.1. Expansion and Contraction of Detoxification Genes in S. chinensis

The sequenced genomes of *A. pisum*, *M. persicae,* and *C. cedri* with manually annotated detoxification gene families served as comparisons for our studies [50,51]. The number of detoxification genes in *C. cedri* was derived from the annotation table in NCBI with Accession No. GCA_902439185.1, and those of *A. pisum* and *M. persicae* were derived from published articles [16,52,53,54].

The number of detoxification genes of *S. chinensis* was 144, less than that of *A*. *pisum*, *C. cedri* and *M. persicae*, which might be related to its unique host plant. It was predicted that polyphagous insects require a greater complement of detoxification-related enzymes for they were usually exposed to a higher diversity of plant secondary metabolites than oligophagous ones [54,55]. The gene number of GSTs and UDPs in *S. chinensis* was nine and 14, respectively, which were relatively less and conserved by comparison with the other three aphids. The fewer detoxification-related genes may be due to the unobvious duplication events of detoxification-related genes. Genes with conserved roles usually have relatively stable copies, while those with diversified functions have higher rates of gain-and-loss with random changing degrees [56]. The numbers of ABCs of *S. chinensis* was 55, that was more than other aphids and showed significant genetic expansion. It is inferred that the ABCs play an important role in the degradation of secondary metabolites of *S. chinensis*. The number of P450s was 48 in *S. chinensis*, which was clearly contracted by comparison with the other three aphids. CYPs in many insects are associated with the metabolism or detoxification of key endogenous substrates and xenobiotics, such as steroid hormones and lipids, plant natural products, and pesticides, which are key components for the successful adaptation of insects to their host plants [57].

### 4.2. Characteristics and Expression of the Detoxification Genes of S. chinensis

The current study compared characteristics of the five detoxification gene families of *S. chinensis* with other three aphids, *A. pisum*, *M. persicae*, and *C. cedri*. Ten conserved motifs were found in four gene families, except for GSTs with six conserved motifs. The conserved motifs in detoxification genes are very important in the functional domains, and the highest one was its key structure, while the motif patterns can finely tune the function of detoxification genes [58]. Structural variation affects gene evolution [59]. The detoxification gene proteins encoded by the subfamily members usually had the same motif orders and exons, which indicated that genes in closely related groups were highly conserved and might have similar functions.

### 4.3. Collinearity, Chromosome Position and Evolutionary Rate of Detoxification Genes of S. chinensis

We performed analysis on collinearity relationships to further investigate the gene duplication events within detoxification genes. There were two pairs of gene duplications in the detoxification genes of *S. chinensis*, including P450s and ABCs. The differential expansion events during the species evolution might result in the phenomena that the number of family members was not correlated with genome size. Tandem duplications generate a large number of genes, which is considered as the most effective mechanism for producing and maintaining gene copies [57]. It was reported that gene duplications were critical for the evolution of new genes and novel functions and were major forces driving gene family expansion [60]. For example, CYP genes, often clustering in genomes, were considered as a result of gene duplication events [56]. We calculated the Ka and Ks values to estimate the evolutionary trend and revealed a functional selection pressure between duplicated gene pairs. The ratio of Ka to Ks in protein-coding genes can determine whether there is selection pressure acting in the process of gene evolution. Detoxification genes of *S. chinensis* showed a strong purifying selection during evolution, which suggested that their functions may be evolutionarily conserved.

## 5. Conclusions

Here, we performed a comprehensive genome-wide analysis of the detoxification gene family in *S. chinensis* and compared the results with the genomes of *A. pisum*, *M. persicae,* and *C.cedri.* We manually annotated 144 genes of *S. chinensis,* including nine in GSTs, 55 in ABCs, 18 in CCEs, 48 in P450s, and 14 in UDPs. We constructed the phylogenetic trees, motif patterns, domains, and gene structures of detoxification genes from these four aphids and further analyzed the chromosomal location, collinearity, evolution rates, and their expression. Finally, we predicted characteristics, physicochemical properties, and protein multi-level structures of detoxification gene products of *S. chinensis*. Our results provide comprehensive information, molecular data, and gene candidates for further analyses. *S. chinensis* can survive in galls with the tannin content up to 70%, and we infer that it has a strong ability to reduce secondary metabolites. The phenomenon may be related not only to its own detoxification genes, but also to the existence of endosymbionts or a long-term obligate parasitic relationship.

## Figures and Tables

**Table 1:** Comparison of detoxification gene numbers in annotated insect species.

	Species	*S. chinensis*	*A. pisum*	*C. cedri*	*M. persicae*
	delta	2	8	3	3
	Sigma	5	5	6	6
GST	Theta	2	1	1	1
	other	0	6	1	1
	Microsomal	0	2	3	2
	Total	9	22	14	13
ABC	A	5	5	5	1
	B	5	3	5	3
	C	15	1	1	0
	D	2	2	2	2
	E	1	1	1	1
	F	3	4	3	3
	G	24	22	33	13
	Total	55	38	50	23
CCE	Venom	3	2	3	4
	Esterase	6	18	12	20
	Acetylcholinesterase	2	8	3	2
	neuroligins	5	4	11	5
	Pyrethroid	2	2	0	2
	Acyl-protein	0	0	3	0
	Pancreatic	0	0	2	0
	Total	18	34	34	33
CYP	CYP2	15	10	14	10
	CYP6	5	23	7	25
	CYP4	27	23	38	23
	mito	1	8	1	7
	Total	48	64	60	65
UDP	UGT4	7	8	8	10
	UGT5	7	10	9	10
	Total	14	18	17	20
	Total detoxification genes number	144	176	175	157

Notes: Venom present Venom carboxylesterase; Pyrethroid present Pyrethroid hydrolase Ces2e; Acyl-protein present Acyl-protein thioesterase; Pancreatic present Pancreatic triacylglycerol lipase.

**Table 2:** Nucleotide substitution rate of detoxification genes in *S. chinensis*.

Chromosome	Gene ID	Chromosome	Gene ID	Gene Family	Ka	Ks	Ka/Ks
ChrA1	Apis007359	LG01	Schi01G019250	P450s	0.0960	1.1974	0.0802
ChrA1	Apis008190	LG01	Schi01G019490	UDPs	0.1782	1.1658	0.1529
ChrA1	Apis008119	LG01	Schi01G012970	UDPs	0.1767	0.6031	0.2930
ChrA1	Apis004941	LG01	Schi01G032520	UDPs	0.2523	1.2278	0.2055
ChrA1	Apis007513	LG01	Schi01G006260	ABCs	0.1484	1.0116	0.1467
ChrA1	Apis006331	LG05	Schi05G007220	GSTs	0.1966	1.0702	0.1837
ChrA1	Apis005733	LG05	Schi05G006460	ABCs	0.3801	1.5960	0.2382
ChrA1	Apis007503	LG06	Schi06G007150	ABCs	0.1901	0.7266	0.2616
ChrA1	Apis007306	LG06	Schi06G004390	GSTs	0.0826	3.3468	0.0247
ChrA1	Apis008003	LG06	Schi06G010000	P450s	0.1018	0.7825	0.1301
ChrA1	Apis005719	LG06	Schi06G003040	P450s	0.1275	2.1154	0.0603
ChrA1	Apis005195	LG06	Schi06G009880	ABCs	0.0255	1.2537	0.0203
ChrA2	Apis009460	LG04	Schi04G003120	P450s	0.1320	1.2539	0.1053
ChrA3	Apis010999	LG01	Schi01G040830	P450s	0.1155	1.2887	0.0897
ChrA3	Apis015091	LG01	Schi01G040920	P450s	0.2035	1.2475	0.1631
ChrA3	Apis014264	LG01	Schi01G008900	ABCs	0.1457	1.6398	0.0888
ChrA3	Apis013170	LG01	Schi01G030540	UDPs	0.0639	0.8659	0.0738
ChrA3	Apis011342	LG02	Schi02G004480	CCEs	0.4770	1.6018	0.2978
ChrA3	Apis011920	LG02	Schi02G004050	P450s	0.0970	1.0765	0.0901
ChrA3	Apis013813	LG02	Schi02G004380	UDPs	0.0807	0.7942	0.1017
ChrA3	Apis016003	LG02	Schi02G008970	ABCs	0.0530	0.7650	0.0693
ChrA3	Apis012170	LG02	Schi02G005610	ABCs	0.0683	0.6467	0.1055
ChrA3	Apis015017	LG02	Schi02G004480	CCEs	0.0121	0.5458	0.0222
ChrA3	Apis013379	LG03	Schi03G008990	ABCs	0.0307	0.8186	0.0375
ChrA3	Apis015108	LG03	Schi03G001540	ABCs	0.0462	0.9221	0.0501
ChrA3	Apis013979	LG03	Schi03G003720	UDPs	0.3973	1.2232	0.3248
ChrA3	Apis015779	LG03	Schi03G000690	GSTs	0.2557	2.9061	0.0880
ChrA3	Apis013557	LG03	Schi03G001480	ABCs	0.1222	1.6464	0.0742
ChrA3	Apis013139	LG03	Schi03G001540	ABCs	0.1233	1.8349	0.0672
ChrA3	Apis015626	LG03	Schi03G003720	UDPs	0.0960	1.1974	0.0802
ChrA3	Apis012434	LG04	Schi04G000750	ABCs	0.1782	1.1658	0.1529
ChrA3	Apis013152	LG04	Schi04G008270	ABCs	0.1767	0.6031	0.2930
ChrA3	Apis016040	LG05	Schi05G004480	ABCs	0.2523	1.2278	0.2055
ChrA3	Apis013491	LG05	Schi05G003020	ABCs	0.1484	1.0116	0.1467
ChrA3	Apis013103	LG05	Schi05G003270	CCEs	0.1966	1.0702	0.1837
ChrX	Apis002253	LG08	Schi08G001700	P450s	0.3801	1.5960	0.2382
ChrX	Apis002712	LG08	Schi08G002470	P450s	0.1901	0.7266	0.2616
ChrX	Apis003375	LG08	Schi08G003750	ABCs	0.0826	3.3468	0.0247

**Table 3:** Expression profiles of detoxification genes in *S. chinensis*.

Gene Family	Gene ID	Transcriptome ID	A4601	A4603	A4621
ABCs	Schi05G003020	TRINITY_DN11865_c0_g1	14.69	31.81	10.8
	Schi01G030320	TRINITY_DN15325_c0_g1	4.37	9.1	3.15
	Schi03G004900	TRINITY_DN1743_c0_g1	58.06	31.76	58.18
	Schi02G007740	TRINITY_DN217_c0_g1	48.23	30.84	44.34
	Schi03G001540	TRINITY_DN3732_c0_g1	45.22	41.05	45.98
	Schi01G023860	TRINITY_DN4691_c0_g1	4.4	11.24	3.2
	Schi08G003750	TRINITY_DN5271_c0_g1	9.21	24.36	6.8
	Schi01G006320	TRINITY_DN6971_c0_g2	12.12	30.29	8.88
	Schi01G030320	TRINITY_DN7197_c0_g1	7.91	19.44	6.43
	Schi02G008970	TRINITY_DN7586_c0_g2	59.9	45.04	36.17
	Schi06G007150	TRINITY_DN761_c0_g1	33.79	52.68	30.66
	Schi03G003460	TRINITY_DN8762_c0_g1	15.04	36.19	10.17
	Schi01G002380	TRINITY_DN9280_c0_g3	98.6	79.59	117.28
CCEs	Schi05G003310	TRINITY_DN204_c0_g1	62.97	68.46	62.34
	Schi02G003120	TRINITY_DN3432_c0_g1	35.65	23.98	36.87
	Schi05G003270	TRINITY_DN400_c0_g2	151.5	142.6	87.47
	Schi05G003280	TRINITY_DN400_c0_g3	16.68	33.05	14.58
	Schi02G008710	TRINITY_DN433_c0_g1	63.77	51.81	44.62
	Schi06G007520	TRINITY_DN5_c0_g1	9.38	6.88	11.28
	Schi02G004480	TRINITY_DN5_c0_g2	67.24	45.68	70.95
	Schi03G008370	TRINITY_DN6210_c0_g1	2.54	4.9	2.21
	Schi01G016690	TRINITY_DN9661_c0_g1	6.44	15.25	3.19
GSTs	Schi01G005590	TRINITY_DN1619_c0_g1	243.24	358.54	153.26
	Schi06G004390	TRINITY_DN1656_c0_g1	66.68	94.35	46.85
	Schi05G002040	TRINITY_DN2301_c0_g1	30.17	36.47	19.08
	Schi05G002060	TRINITY_DN2393_c0_g1	23.44	47.37	20.04
	Schi01G013300	TRINITY_DN4283_c0_g1	10.46	7.58	6.84
	Schi01G013290	TRINITY_DN7068_c0_g1	11.88	7.23	7.74
	Schi01G005590	TRINITY_DN1619_c0_g1	243.24	358.54	153.26
P450s	Schi01G035210	TRINITY_DN12170_c0_g1	1.64	2.36	1.66
	Schi01G003300	TRINITY_DN12662_c0_g1	159.04	172.47	169.26
	Schi05G007430	TRINITY_DN13254_c0_g1	3.1	9.25	0
	Schi06G002630	TRINITY_DN13524_c0_g2	5.09	9.8	3.91
	Schi04G003130	TRINITY_DN1463_c0_g1	27.16	46.06	29.89
	Schi05G007400	TRINITY_DN1506_c0_g1	45.49	15.18	22.98
	Schi06G003040	TRINITY_DN18244_c0_g1	2.71	5.45	1.97
	Schi05G007440	TRINITY_DN19060_c0_g1	0	8.66	0
	Schi06G003050	TRINITY_DN2747_c0_g1	71.64	22.18	57.66
	Schi01G040920	TRINITY_DN3861_c0_g1	6.54	6.95	5.63
	Schi01G031590	TRINITY_DN4207_c0_g1	21.92	8.28	23.68
	Schi06G008170	TRINITY_DN4410_c0_g1	5.75	20.11	4.91
	Schi08G004540	TRINITY_DN6310_c0_g1	11.6	26.8	9.39
	Schi02G004050	TRINITY_DN7229_c0_g1	11.07	13.29	4.56
	Schi06G010020	TRINITY_DN7575_c0_g1	4.26	0.54	0.4
	Schi06G010020	TRINITY_DN7575_c0_g3	4.73	0	0
	Schi01G040830	TRINITY_DN9199_c0_g1	1.92	0.19	1.37
	Schi08G002470	TRINITY_DN9598_c0_g2	112.17	167.03	51.8
	Schi04G003120	TRINITY_DN9796_c0_g1	41.5	74.21	39.17
UDPs	Schi01G019500	TRINITY_DN10423_c1_g1	1.26	3.27	0.94
	Schi01G039250	TRINITY_DN12730_c0_g2	1.31	9.08	5.64
	Schi01G040430	TRINITY_DN13038_c0_g1	2.31	1.89	1.81
	Schi01G039420	TRINITY_DN1305_c0_g2	3.84	2.98	1.74
	Schi03G003710	TRINITY_DN18843_c0_g1	146.64	80.5	57.87
	Schi01G012970	TRINITY_DN2128_c0_g1	22.23	23.55	22.69
	Schi03G009580	TRINITY_DN317_c0_g1	15.2	12.18	9.12
	Schi01G001200	TRINITY_DN3281_c0_g1	4.04	11.34	3.64
	Schi03G003720	TRINITY_DN7236_c0_g1	4.15	4.55	6.76
	Schi01G030560	TRINITY_DN8152_c0_g1	64.69	106.98	50.32
	Schi01G030530	TRINITY_DN8293_c0_g1	9.97	15.36	10.36

**Table 4:** General information and physicochemical properties of detoxification gene family products in *S. chinensis*.

Sequence ID	Number of Amino Acid	Molecular Weight	Theoretical pI	Instability Index	Aliphatic Index	Grand Average of Hydropathicity
Schi01G002380	593	65274	9.14	38.36	93.19	−0.032
Schi01G004180	697	78885.97	8.01	43.68	87.99	−0.105
Schi01G006260	613	69226.58	8.24	34.52	104.44	0.062
Schi01G006320	1764	199951.12	6.69	32.6	100.96	0.084
Schi01G008900	498	57553.93	8.99	40.15	99.04	−0.086
Schi01G014120	840	95851.13	6.94	33.89	112.86	0.279
Schi01G023860	1499	170713.27	8.49	36.64	109.17	0.231
Schi01G030320	641	71130.22	9.58	35.86	110.11	0.168
Schi02G005610	908	100248.13	8.01	49.66	95.9	0.071
Schi02G007740	1521	167840.78	6.38	41.08	89.57	−0.134
Schi02G008970	1293	142261.06	6.3	34.07	91.01	−0.01
Schi03G001480	684	76921.85	6.71	41.96	99.37	0.186
Schi03G001540	1635	185425.46	8.04	35.94	90.94	0.017
Schi03G002100	686	77033.25	9.02	41.24	96.47	0.054
Schi03G003460	585	66430.83	8.85	35.58	105.85	0.306
Schi03G003470	601	69150.27	8.86	28.79	106.67	0.192
Schi03G003490	600	68562.17	8.96	36.69	100.35	0.149
Schi03G003540	617	69493.45	8.19	32.78	100.29	0.212
Schi03G004900	629	71054.84	8.31	42.09	92.05	0.1
Schi03G008990	640	71570.56	7	39.29	95.33	0.097
Schi04G000750	633	71548.71	8.55	36.92	94.04	0.085
Schi04G008270	685	76112.09	8.65	33.16	93.18	0.053
Schi05G003020	608	68690.7	8.34	35.97	95.21	−0.235
Schi05G004480	905	101044.93	8.95	49.4	91.8	−0.127
Schi05G004490	639	71771.98	8.74	39.2	92.35	0.085
Schi05G006460	693	77850.75	6.83	45.54	96.44	0.101
Schi06G004340	620	69928.82	8.79	29.38	112.35	0.278
Schi06G007150	664	75912.44	9.47	33.85	95.99	−0.088
Schi06G009880	612	69573.74	6.58	43	105.23	0.053
Schi06G009890	760	84442.58	6.55	48.21	101.42	0.219
Schi07G000280	685	76153.91	7.76	42.6	106.98	0.237
Schi08G003750	952	107624.75	5.34	39.13	82.31	−0.81
Schi01G016690	821	93445.09	6.75	40.89	76.86	−0.355
Schi01G037470	572	64581.95	6.04	38.14	84.34	−0.148
Schi02G003120	819	89348.73	8.2	38.23	76.96	−0.344
Schi02G004480	1185	129059.87	8.96	47.73	74.78	−0.481
Schi02G008710	604	68570.23	6.13	41.21	82.65	−0.219
Schi05G003270	571	64053.44	6.03	39.38	78.83	−0.194
Schi05G003280	562	63291.26	5.78	44.64	82.19	−0.161
Schi05G003290	544	61533.78	6.11	45.8	82.78	−0.218
Schi05G003300	580	64668.35	5.77	33.81	84.52	−0.097
Schi05G007230	216	24128.85	6.1	20.08	98.01	−0.103
Schi01G005590	203	23406.02	5.19	31.63	98.47	−0.164
Schi03G000690	178	20632.58	5.96	31.48	93.54	−0.358
Schi06G004390	239	27282.58	6.44	41.82	80.84	−0.204
Schi05G002060	232	27348.65	7.61	49.68	88.62	−0.342
Schi01G013290	203	23437.29	5.69	41.89	106.65	−0.055
Schi03G000710	200	23003.31	6.22	27.1	92.05	−0.277
Schi05G007220	245	27570.38	5.96	39.51	92.73	−0.149
Schi01G013300	199	22898.4	4.93	48.42	98.94	−0.091
Schi05G002040	232	27362.88	8.79	46.39	94.57	−0.399
Schi01G003300	499	57013.79	6.17	41.35	86.33	−0.179
Schi01G019250	511	58805.92	8.65	41.65	91.94	−0.188
Schi01G019260	524	59948.91	8.8	35.91	80	−0.262
Schi01G031590	507	57702.75	7.52	40.12	83.27	−0.117
Schi01G035210	396	45966.59	9.04	45.29	95.96	−0.252
Schi01G040830	507	58238.17	8.57	43.6	88.58	−0.143
Schi01G040920	560	62588.1	7.94	45.02	88.12	−0.093
Schi02G004050	488	56109.11	9.07	34.91	96.29	−0.134
Schi04G003120	528	61269.32	8.84	47.3	86.93	−0.357
Schi04G003130	518	60267.63	9.17	46.5	89.02	−0.402
Schi06G002630	476	54574.68	9.07	37.99	100.53	−0.026
Schi06G003040	497	56659.03	6.2	43.24	97.71	−0.022
Schi06G003050	511	58396.33	6.63	39.11	84.5	−0.268
Schi06G008170	533	61800.84	8.66	47.71	92.51	−0.211
Schi06G010000	511	58109.91	8.06	48.69	93.27	−0.18
Schi06G010010	650	73866.86	9.14	43.18	92.26	−0.238
Schi06G010020	526	60232.45	8.65	41.22	96.9	−0.196
Schi08G001700	317	37661.5	5.85	43.89	92.43	−0.309
Schi08G002470	503	57336.76	8.29	41.37	103.34	−0.069
Schi08G004540	517	60112.02	8.86	44.51	86.91	−0.142
Schi01G012990	539	61628.03	8.45	41.01	94.55	−0.074
Schi01G030560	501	56860.86	9.43	34.5	98.82	0.049
Schi01G012970	531	59853.37	8.87	50.01	92.86	−0.025
Schi01G032520	523	57472.95	8.77	34.76	87.59	0.044
Schi01G039280	520	59713.36	8.47	32.2	91.98	−0.056
Schi01G040430	509	56991.26	8.24	33.94	99	0.148
Schi01G030550	522	60046.73	8.74	33.29	91.99	−0.012
Schi01G019490	980	112852.53	8.53	44.63	101.61	0.054
Schi01G030540	469	50823.23	7.26	47.68	88.64	−0.023
Schi03G003720	500	57925.31	6.01	47.58	96.64	0.023
Schi01G039290	516	58981.55	7.31	31.68	97.05	0.041
Schi01G039250	493	56824.73	6.64	40.2	97.26	0.045
Schi02G004380	492	56852.92	8.46	39.2	92.09	−0.073
Schi01G030530	503	57393.02	8.44	35.94	91.63	−0.094
Schi02G001900	521	58931.78	8.83	40.85	96.74	0.07
Schi01G012980	540	62271.05	7.32	43.43	92.59	−0.069

**Table 5:** Secondary structure of the GST gene family proteins in *S. chinensis*.

Protein	α Helix (%)	Extended Strand (%)	β Turn (%)	Random Coil (%)
Schi05G007230	49.54	13.43	6.94	30.09
Schi01G005590	53.69	8.87	3.94	33.5
Schi03G000690	58.43	6.74	5.06	29.78
Schi05G002060	50.43	9.05	5.17	35.34
Schi01G013290	51.23	11.33	4.43	33
Schi03G000710	50.5	9.5	4	36
Schi05G007220	45.71	16.73	6.94	30.61
Schi01G013300	55.78	11.06	4.02	29.15
Schi05G002040	52.59	7.33	5.17	34.91

## Data Availability

The protein coding sequences of detoxification genes of the three aphid species, *Acyrthosiphon pisum*, *Cinara cedri* and *Myzus persicae*, were downloaded from the Insect BASE website (http://v2.insect-genome.com/, accessed on 12 May 2022).
